# New insight into DAVF pathology—Clues from meningeal immunity

**DOI:** 10.3389/fimmu.2022.858924

**Published:** 2022-09-15

**Authors:** Tianqi Tu, Zhenghong Peng, Zihao Song, Yongjie Ma, Hongqi Zhang

**Affiliations:** ^1^ Department of Neurosurgery, Xuanwu Hospital, Capital Medical University, Beijing, China; ^2^ International Neuroscience Institute (China-INI), Xuanwu Hospital, Capital Medical University, Beijing, China; ^3^ Department of Health Management Center, The Affiliated Hospital of Southwest Medical University, Luzhou, China

**Keywords:** meningeal immunity, meningeal lymphatic vessels, meninge barrier, dural sinus, dural arteriovenous fistula (DAVF)

## Abstract

In recent years, with the current access in techniques, studies have significantly advanced the knowledge on meningeal immunity, revealing that the central nervous system (CNS) border acts as an immune landscape. The latest concept of meningeal immune system is a tertiary structure, which is a comprehensive overview of the meningeal immune system from macro to micro. We comprehensively reviewed recent advances in meningeal immunity, particularly the new understanding of the dural sinus and meningeal lymphatics. Moreover, based on the clues from the meningeal immunity, new insights were proposed into the dural arteriovenous fistula (DAVF) pathology, aiming to provide novel ideas for DAVF understanding.

## 1. Brain and its immune protection

The central nervous system (CNS) of humans has evolved in an extremely complex way, which enables it to become an intelligent organ to receive multiple input sensory and then integrate these signals and feedback appropriate outputs, achieving precise control of the overall behavior activities (1, 2). For decades, the understanding from “The brain is an immune-privileged organ” to “The meningeal immune system plays critical roles in maintaining brain function” continues to be refined (3, 4). It is not surprising that the most complex organ of the human body is constantly exposed in the antigen contexts. Due to the extraordinary strength of the protection of the meningeal immune system (MIS), which maintains the physical cushion and immune protection against injurious impacts, various physiological activities of the CNS can proceed normally without interference (5). Meanwhile, accumulating evidence reminds us that meningeal immunity may represent origins for numerous neurological disorders (3, 6, 7).

## 2. Meningeal immune system

For centuries, the only functional significance of the elaborate barrier system was considered to physically protect the CNS, and the brain was once thought to be an immunity-privileged organ (8, 9). In recent decades, researchers in this field have made great contributions to reveal that the roles of immune surveillance and response could also be performed by the meningeal barrier system (4). On the macro level, the MIS consisted of meninges (a multiple-layer membrane including the dura, arachnoid, and pia mater), meningeal vasculature (meningeal arteries, venous sinuses, and lymphatic vessels), and meningeal nerve branches (10–13). From the micro space, the neuro-immune cell unit is the basis of the system, running the interaction between the CNS and the immune system (14–17). Cytokines and related immune molecules are the major executives to steer and generate immunoregulatory responses (18, 19). The following descriptions will highlight the important functions of each component.

### 2.1 The meningeal barrier

Diverse innate and adaptive immune mechanisms in CNS borders have been described recently (13, 20, 21). Interestingly, the meninges are ideal options to explore the CNS immune surveillance. Collectively known as the meninges, the membranous structures on the surface of the brain are made up of three layers of cellular-layered membranes.

The outermost layer adjacent to the skull is the dura mater, which could be distinguished into the periosteal layer and the meningeal layer (8). The dura mater could perform efficient immune surveillance by driving immune cell circulation and maintaining infiltration homeostasis (22, 23). The dural sinus is the characteristic structure of the dural vascular architecture. Notably, potent evidence revealed it could be a neuroimmune interface, which enables CNS immune surveillance. In the presence of neuroinflammatory infiltration, the immune niche will be altered, leading to the appearance of related pathological features.

The innermost layer adhered tightly to the surface of the cerebral and spinal parenchyma is the transparent pia mater. This layer is semipermeable to the cerebrospinal fluid (CSF) that flows into perivascular spaces. Previous evidence demonstrated that the pial meninges might be the entry point for peripherally immune cells entering into the CNS (4, 8). The middle layer is the peculiar spiderweb-like membrane, which is a tight arachnoid barrier, regulating the transportation of molecules by tight junction-like structures. Anatomically, it separates the dura layer from the CNS parenchyma. Collectively, the pial membrane and arachnoid are called leptomeninges (8).

**Figure 1:**
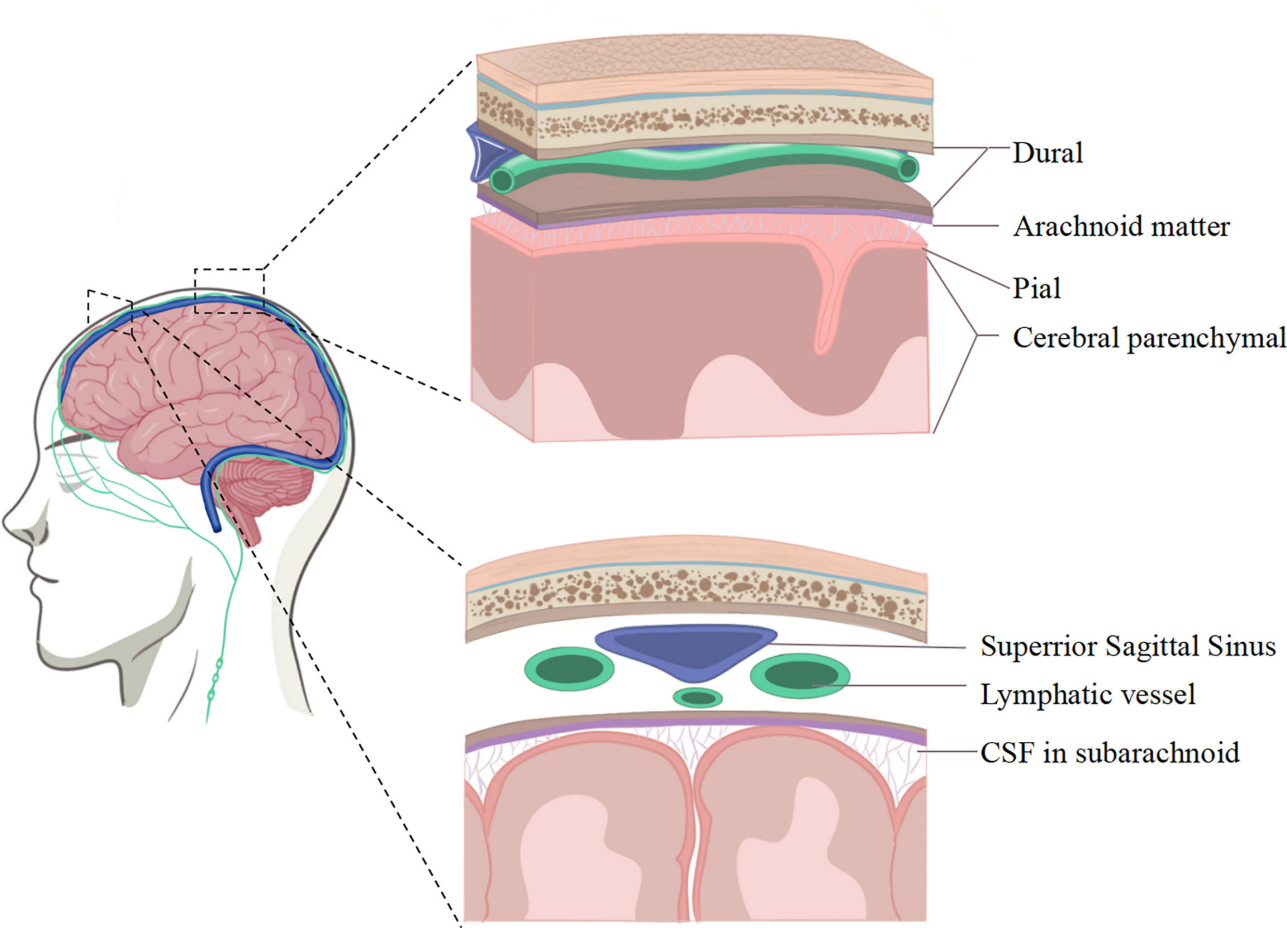
Illustration of the meningeal immune system.

Housed between pial and arachnoid meninges, the CSF flows smoothly, ensuring the normal circulation of necessary substances and the metabolism of various accumulation. Meanwhile, the buoyancy provided by the fluid is an artful force to respond to impact or squeeze from its own mass or movement. The three meninges are separated but cooperate together, participating in the CNS immune surveillance (**Figure 1**).

### 2.2 The meningeal vasculature

The meninges are highly vascularized, and the structure and function of the vascular architecture are complex. In this part, the emphasis is placed on the description of meningeal lymphatics and dural vessels and sinuses to fully understand their structure and newly recognized functions.

#### 2.2.1 Meningeal lymphatic vessels: From the past to the updated view

Centuries ago, the Italian anatomist Paolo Mascagni described the anatomical discoveries of meningeal lymphatic vessels (MLVs) exactly. However, controversies about the lack of lymphatic circulation in the cerebra ensued (24). After nearly 200 years of debate, the advanced imaging techniques provide definitive structural and functional characterization of MLVs (12, 25–29). More importantly, since CNS interaction with peripheral immunity has been firmly established due to MLV linkage, the concept of immune privilege in the CNS is completely broken (3–5).

In most species, such as fish, rodents (rats and mice), nonhuman primates, and even humans, meningeal lymphatics are found to be evolutionarily conserved (29–32). Previous findings reported that MLVs were paralleled to the dural venous structures. Recent publications refreshed the findings: the meningeal lymphatics at the lateral and basal skull creep with the cranial nerves’ track and then pass through the skull and drain to cervical lymph nodes (CLNs) (30, 33). Although the exact transportation line of materials from the parenchyma and CSF is still in debate, some results suggested that these vessels could directly connect the CNS to the peripheral immune system *via* draining immune molecules and cells into the deep CLNs (26).

The existence of the meningeal lymphatics is a landmark discovery that opened the way to current research of meningeal immunity. Recent works supplied comprehensive insights about the meningeal lymphatics’ function during homeostasis, aging, and even in CNS disorders (26, 28, 34–36). The CSF, the interstitial fluid (ISF), and the lymphatic fluid are important media for the circulation of immune cells and molecules. The application of tracers allows us to visualize the immune trajectories, which is a complex process related to numerous impact factors including arterial pulsation and the fluid pressure of each part (33, 37). The currently accepted path is as follows: endogenous antigens (metabolites and proteins) from the cerebral parenchyma are presented into the ISF, subsequently entering the subarachnoid space, then drained by lymphatic vessels to the CLNs. Future observations will present convincing arguments that meningeal lymphatics are important outflows of CSF molecules.

Recent works of Chen et al. (38–40) are groundbreaking. In 2019, they showed that after cerebrovascular injury, the meningeal lymphatics could grow into the damaged brain. As a response of repair, the ISF would be drained by ingrown lymphatics to relieve cerebral edema (38). Meanwhile, as “growth tracks,” neovascularization can be “guided” by the lymphatic vessels to complete reconstruction, and then the ingrown lymphatic vessels will disappear in the form of apoptosis (38). Two years later (in 2021), a further study added brilliance to the present splendor. The results showed that by direct transdifferentiation of ingrown lymphatic vessels, acute cerebrovascular regeneration could also be achieved (39). Furthermore, according to their latest study (in 2022), the molecular mechanism about how the vessel directionality of cerebrovascular regeneration was revealed, which was closely related to the Cxcl12b/Cxcr4a signal pathway (40). These findings highlighted the involvement of meningeal lymphatics in the regeneration or neovascularization after vascular injury, which undoubtedly opened up a new world for the research of cerebrovascular diseases. In line with the above evidence, it is reasonable to reconsider whether the pathogenesis of the dural arteriovenous fistula (DAVF) formation is related to MLVs.

#### 2.2.2 Dural sinus “newly discovered“: More than just a drainage channel

Normally, the main blood supply to the dura comes from the external carotid artery (ECA) system, and venous blood is collected through the sinus and enters the venous system. The fenestrated vasculature is a characteristic of the dural vascular architecture (8, 11, 22). Recent findings challenged the traditional concept: dural sinus is more than just a drainage channel but may serve as a neuroimmune interface (23). Briefly, these breakthroughs could be described as follows: 1) The CNS-derived antigens in the CSF are found to accumulate at dural sinuses. 2) Captured by dural sinus-associated antigen-presenting cells (APCs), these antigens would be presented to patrolling T cells. 3) T cell trafficking could be orchestrated by the dural sinus stroma. In turn, by recognizing antigens from the CSF, T cells could also enhance the phenotype and effector function of tissues in the dura mater.

Similarly, accumulative works revealed that immune cells such as T cells and APCs are present in these immune synapses to perform immune surveillance (41–43). More importantly, although disease outcomes vary, the initial process of triggering an immune response may be consistent, taking the activation of APC–T cell interaction as an example (23, 41–43). Fitzpatrick et al. (20) added to the knowledge of dural sinus from meningeal humoral immunity. Adjacent to dural venous sinuses, the IgA-secreting plasma cells were observed on the meninges during homeostasis. In the condition of aging and neuroinflammation, it is also proven that this vulnerable venous barrier surface is essential for defending the CNS. Thus, exploring the relationship between the dural sinus immune function and the (patho)physiological process of neurological diseases is promising.

#### 2.2.3 The vasculature-associated barriers: Special shield for CNS parenchyma

The vasculature-associated barriers are of significance to the traditional concept of CNS immunity. In addition to the physical protection of meninges, the vasculature-associated barriers, involving the blood–brain barrier (BBB) and blood–meninge barrier (BMB), also provide special protection for the CNS parenchyma. BBB is a specialized vasculature barrier with selective impermeability, which allows for partially blood-derived products to enter the parenchyma (44–46). Similarly, the BMB is also a specialized selective impermeability barrier, preventing the entrance of blood components from the leptomeningeal vasculature into the CNS (4, 47). Under pathological conditions, the previously impermeable blood-derived factors may enter the brain parenchyma, causing a cascade of reactions including immune responses (45, 46).

Notably, nonselective leakage of blood components from the CNS vasculature, which is the main result of the BBB damage, is frequently observed in diverse acute or chronic neurological disorders (48, 49). The primary and/or secondary immune responses and associated inflammation are increasingly recognized mediators of diverse cerebral anomalies. Immune and inflammation responses precipitate in the BBB breakdown, dysregulated transport, and immune trafficking, aggravating endothelial dysfunction. Importantly, these events also happen to link immune interactions with cerebrovascular dysfunction. In addition, the localized vascular anomalies, such as the DAVF, exactly occurred in meningeal vasculature. Consequently, it is reasonable to believe that the pathological processes including the formation and development of DAVF are closely related to meningeal immunity.

### 2.3 The meningeal innervation

The meninges are highly innervated by various nerve fibers, involving sympathetic and parasympathetic nerves, as well as sensory fibers (50–53). Many pioneers have contributed to the description of the meningeal innervation, revealing that there are significant differences in innervation between the pachymeninx and the leptomeninx of the cranial cavity and spine (4, 54).

The leptomeninx innervation is mainly focused on the cerebral arteries. Fricke et al. (55) researched the localization of nerve fibers in the ventral leptomeningeal connective tissue compartment and described the nerve fibers within the pia that appear to be the primary termination. It must be noted that differentiated innervation patterns of the leptomeninx were also presented in their work in 1997 (55).

Andres et al. (56) reported that the innervation of the dura mater is also extensive, and the vascular and nonvascular regulation targets are both related to the dural fibers. Various segments of the meningeal vessels and venous sinuses are the main location (56).

In addition, the vasoconstrictor and the associated blood flow could be regulated by neuropeptides (50, 57–60). Peripheral nerve fibers could also project to the meninges (60, 61). For providing important sensory feedback, sensory fibers that control changes in temperature, pH, and mechanical pressure extend within the meninges (62, 63). Most importantly, like the skin barrier and gastrointestinal mucosa, numerous immune hotspots and messages could also be found around dural sinuses (4, 64, 65). The above evidence feasibly intrigued our interests to investigate the roles of meningeal innervation in maintaining meningeal immunity homeostasis.

### 2.4 The meningeal cells, molecules, and immunity function

Immune cells and related characteristic cytokines are the primary guardians of immune homeostasis, which actually execute all immune regulation instructions. Cellular immunity and humoral immunity are essential for the protection of the CNS parenchyma. For decades, the spectrum of these microscopic molecules’ roles in physiological and pathological conditions gradually becomes clear.

#### 2.4.1 The meningeal cellular immunity

So far, accumulated evidence built the intact framework of the meningeal cellular immune system, and main studies were focused on T cells and macrophages (26, 64, 66–69). The meningeal T cell always acts as the immune surveillance guard and immunohomeostasis maintainer (12, 26, 43, 68–72). As the latest findings we mentioned above, the CNS-derived antigens accumulated around the dural sinuses could be captured by APCs and then be presented to patrolling T cells through the CSF pathway (23). Activated T cells would promote meninges to initiate antigen processing. In the research field of multiple sclerosis (MS), studies on meningeal T cells provided a new consideration of neuroimmunology and the MS pathology. As MS is believed to be mediated by myelin-specific T cells, the potential mechanisms of how T cells acquire their encephalitogenic phenotype and initiate inflammation have been demonstrated (26, 43, 68, 69). In addition, when searching for the pathway of how the T cells reach the CSF and get into and out of the meninges, the functional lymphatic vessels lining the dural sinuses were discovered (12, 71). The T cells were also shown to have the potential to support memory and learning, which may shed light on immune-based therapy exploration for cognitive decline (72). In the experimental models of autism and aggregation behavior, meningeal γδ17 T cells were suggested to promote behavioral changes (70). These findings suggested that meningeal T cells are potent executors of meningeal immune function.

Specialized macrophage populations at the CNS borders perform critical roles in health and disease conditions (64). As the most abundant cell population in healthy meninges, macrophages were reported to form the origin to the phenotype turnover (73, 74). The meninge-associated macrophages could be divided into three populations: leptomeningeal macrophages, perivascular macrophages, and choroid plexus macrophages (64, 73). To date, all of these categories are thought to guard the meningeal barriers and to control the antigens and metabolite drainage (64, 73, 74). In the MS research field, activated macrophages are found abundantly accumulated before clinical symptoms caused by inflammatory infiltration (75, 76). In neurodegenerative diseases, meningeal macrophages are reported to have dual roles (31, 35, 77, 78). By removing insoluble amyloid-β, macrophages could help in delaying or salvaging cerebral amyloid angiopathy (79). However, once overburdened, dysfunctional macrophages would act as internal hostile molecules to exacerbate Alzheimer’s disease (AD) pathology processes (79, 80).

Notably, the brain borders are inundated with highly populated immune cells, and multiple immune cell subsets coordinate with each other to maintain normal immune function (81).

#### 2.4.2 The meningeal humoral immunity

In recent years, one of the major breakthroughs in the research field of the MIS is undoubtedly the supplementation of meningeal humoral immunity knowledge (16, 17, 20). Given that studies in recent decades are mainly focused on meningeal cellular immunity, a detailed generalization of meningeal humoral immunity is not available. In this part, we summarized the contents of meningeal humoral immunity comprehensively, providing a new perspective for further research.

B cells have previously been shown to be located near the dural sinus regions with slow blood flow. Once there is fenestration, these blood-borne pathogens could be permitted to access the CNS parenchyma (82). Traditionally, circulating antibodies were not thought to be present in the CNS of healthy individuals. However, in the disease state, antibodies involving IgA and IgG can be found in the CSF (83–85). These pieces of evidence are the location basis for the occurrence of humoral immune events.

In the work of Fitzpatrick et al. (20), they showed that the IgA-secreting plasma cells infiltrated the meninges of humans and mice during homeostasis. With age and disruption of the intestinal barrier, peri-sinus IgA plasma cells increase (20). In addition, meningeal IgA was shown to be essential for defending the vulnerable barrier surface of the CNS in their work (20). Several other outstanding works have clearly described the origin and migration of meningeal B cells (16, 17). The latest findings revealed the following: 1) The meninges host a substantial myeloid cell pool in the brain border, which would mediate immune surveillance. Meanwhile, meninges also harbor a specific lymphopoietic niche for the CNS borders. 2) Under homeostasis, the meningeal immune cells could be supplied by the bone marrow niches adjacent to the brain and spinal cord. 3) Migration of meningeal myeloid cells through the meningeal barrier to parenchyma is the next step in the microenvironment of CNS injury and inflammation (16, 17, 20). Interestingly, through B-cell receptor sequencing, it was also confirmed that the gastrointestinal barrier is an important source of meningeal B cells (20, 86). To understand the origin and the physiological and pathological function of humoral immune cells at the brain border is an essential step in revisiting meningeal immunity, which also encourages the rethinking of therapeutic approaches.

### 2.5 The meninge vessels/innervation cells/molecular units and immunity function

**Figure 2:**
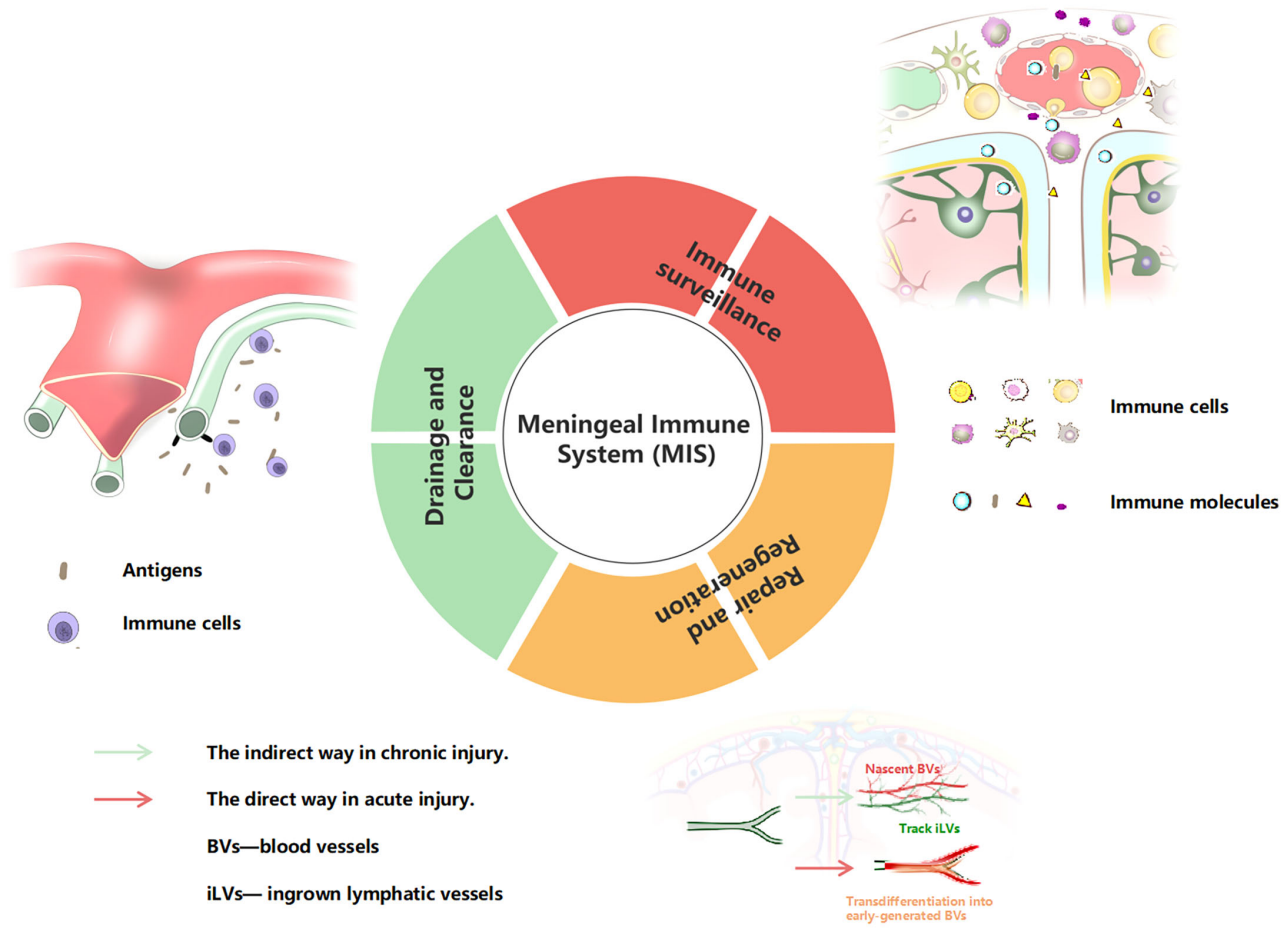
Summary of the MIS functions. BVs, blood vessels; iLVs , ingrown lymphatic vessels.

From what has been reviewed above, we propose the concept of a complete MIS for the first time. The MIS is a tertiary structure. The primary structure is a barrier structure composed of three layers of tangible membrane, involving the dura mater, arachnoid, and pia mater (from outside to inside). The secondary structure is the vasculature and meningeal innervation. The meningeal lymphatics often run parallel to blood vessels, and the dural sinuses are important immunosurveillance sites. The meningeal nerve covers a wide range of areas and plays an important role in regulating vascular function. The third structure is the direct executor of meningeal immune function, including immune cells, and related immune molecules and cytokines, which play ultimate roles both in humoral and cellular immunity. For the function of the MIS, we also clearly summarize it into three aspects: 1) immune surveillance, 2) drainage and clearance, 3) and repair and regeneration (**Figure 2**).

## 3. Meningeal immune system response to central neural system diseases' pathogenesis

The meninges are strategically located in the CNS. In the case of infectious and non-infectious stress, the MIS perceives unbalanced homeostasis earlier and then actively forms an inflammatory response and activates clearance mechanisms before pathogens reach the parenchyma. Abundant pioneering works have repeatedly highlighted the powerful immune function of the meninges (87). In this part, we will briefly summarize the MIS with these diseases characterized by neuroinflammation, then restrict ourselves to discuss the potential possibility of the pathogenesis of DAVF with the meningeal immunity.

### 3.1 General description of the MIS and CNS diseases

**Figure 3:**
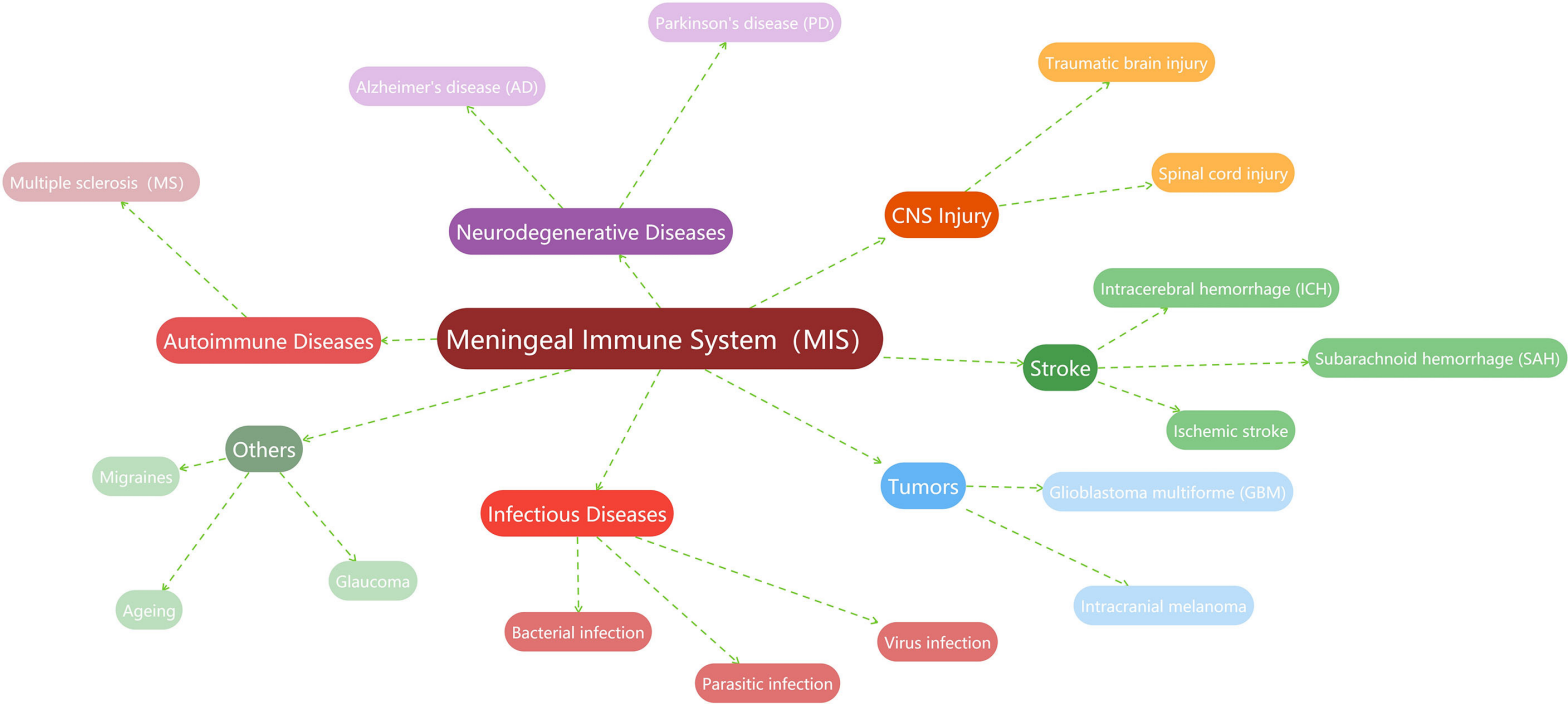
Summary of studies related to the MIS.

In recent years, a vast body of studies has found that the MIS is related to the occurrence, progression, or prognosis of more CNS diseases to varying degrees. For example, the MIS is believed to play important roles in the pathogenesis of multiple sclerosis (26, 88–90), Alzheimer’s disease (35, 91–93), and Parkinson’s disease (78, 94, 95). Meanwhile, the regulation of the MIS about the immune surveillance and lymphatic vessel drainage to the brain tumor is also demonstrated (96–98). Stroke associated with hemorrhage or ischemia is one of the recent research hotspots. Much meaningful work has described the importance of the MIS in cerebral stroke, including subarachnoid hemorrhage (99, 100), intracerebral hemorrhage (101), or ischemic stroke (102, 103). In the brain injury studies, meningeal lymphatic drainage is reported to be associated with prognosis (104, 105). Recently, researchers also found that the MIS has a cross-era function, which is directly or indirectly involved in the repair of cerebrovascular injury (38–40). Naturally, the MIS is linked to the immune responses of infectious diseases, including bacterial meningitis (73) or parasitic infections (69, 106, 107), as well as viral infections (108, 109). In other diseases, such as migraines (110–113), glaucoma (114, 115), and even in the aging process (20, 23, 35), evidence of active immunomodulation and surveillance of the MIS can also be found. Given that this is not the focus of this work, here we summarize these studies related to the MIS in a graph (**Figure 3**).

Of particular note is that the neuroinflammatory condition is an overlapping pathologic process during these disorders, and this happens to be a trigger point for the meningeal immune response (4, 87). These works contributed to prove that the MIS supports normal CNS development and function and governs the pathological process dominated by neuroinflammation (87). It seems that during both noninfectious and infectious microenvironments, neurological dysfunction is often a consequence of meningeal inflammation. Thus, from the view of CNS immune regulation, therapeutic strategies to alleviate these debilitating neurological conditions could be attractive.

### 3.2 New insights into the DAVF pathology

Over the years, researchers have tried to figure out the formation process of vascular abnormalities (116–119). However, it is impossible to know the dynamic formation process because the entity of the lesion is already formed when the patient is presented and diagnosed. Current views tend to define DAVF as an acquired disease, which is closely related to the inflammatory microenvironment after venous hypertension and hypoxia (120–123). Cytokines related to neovascularization are the main pathologic findings. In a 1996 report, researchers examined the angiogenic stimulants in human DAVF sinuses and revealed that the basic fibroblast growth factor (bFGF) levels in the sinus of patients with DAVF were higher when compared with those in the normal group, even in normal dural sinuses (124). Notably, none of the enrolled patients had a history of sinus thrombosis or head injury. Another study confirmed the same results and added that vascular endothelial growth factor (VEGF) was not expressed in normal specimens but was observed in the DAVF group (125).

Certainly, some possible causes as traumatic brain injury (TBI) and sinus thrombosis are thought to be the culprits. It would be reasonable because these injuries could lead to a common pathologic condition—neuroinflammation (122, 126–129). The work of Hamada et al. (130) targeted to discuss this problem. In their research, a comparison was made among five control cases and nine DAVF cases, six of which angiographically showed sinus occlusion. At least in some cases, the venous sinus occlusion might be the cause of hypertension to force the abnormal connections between arteries and veins to open, leading to the formation of DAVFs. At this point, we have reason to postulate that in the inflammatory microenvironment caused by sinus hypertension, the MIS is also responding positively, and the formation of DAVF might be a result of the repair process.

Conclusions drawn in animal models also echoed this (131–140), and one of the outstanding contributions would be attributed to the work of Yang et al. (141). In the context of venous hypertension, the hypoxia and inflammation cascaded, then the infiltration of various angiogenic factors leads to the tendency of abnormal vascular anastomosis. The benign result of venous pressure regress might be related to the formation of compensatory draining veins. Otherwise, development of DAVF and venous hypertension would be the ultimate possible vicious circle. Some of these animal model studies are progressive, while we noticed that the current models do not perfectly reflect the pathophysiology of the disease. Thus, the establishment of stable animal models is urgently needed as well as conducting a series of basic studies to test these hypotheses.

**Figure 4:**
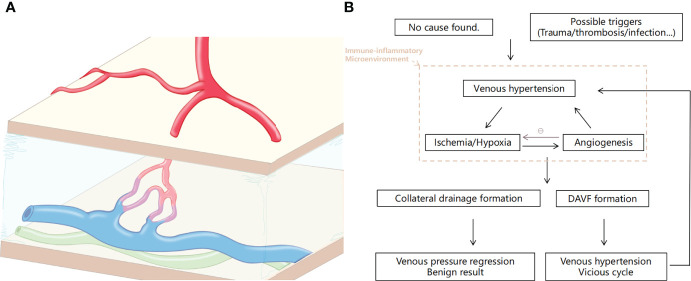
Illustration of the dural arteriovenous fistula (DAVF) and the pathological process of the disease. **(A)** Schematic of DAVF that displayed the abnormal vascular architecture of the dura involving arterial feeders, draining veins, and accompanying lymphatic vessels. **(B)** Brief diagram of the pathophysiological process of DAVF occurrence and development.

Taken together, either as a starting agent or as a result of a vicious cycle, the inflammatory microenvironment is always the background for the occurrence and development of DAVF. The meninges are unlikely to ignore the lesions that occur within themselves. Surely, how this immune reaction works is something fascinating that deserves further study (**Figure 4**).

## 4. Evidence-based assumptions and perspectives

Based on the abundant evidence above, we propose several scientific hypotheses that may open up new directions for future research. Firstly, in the case that the state of the epidural *in situ* or remote site in the CNS is unsteady for a long time, the abnormal vascular anastomoses might be compensatory products of immune surveillance and regulation by the MIS. There are many reasons for this steady-state imbalance, such as trauma, surgery, or infection that can be recorded and observed clearly, while other meningeal immune activation pathways that may have been overlooked, such as a peripheral infection that may communicate with the CNS, are also important mining sites. Secondly, as MLVs have been shown to be involved in the repair and reconstruction of cerebrovascular injury (38, 39), the assumption that the formation of DAVF might be closely related to meningeal lymphatics is quite reasonable. It is not ruled out, and even very likely, that a fistula is one of the imperfect products of the repair process. Thirdly, given the fact that DAVF already exists, the immune monitoring and maintenance functions of the MIS have apparently been activated. A recent work has also discussed cerebrovascular diseases including DAVF from the perspective of CNS immunity, which is undoubtedly a strong support for our assumptions (119). Thus, though it can be challenging, promising alternative therapeutic targets could be explored from the viewpoint of the MIS immune modulation. Excitingly, with the current access as imaging techniques (with tracer agents), gene-edited mice, and high-throughput sequencing, we would be allowed to achieve an unprecedented description of the MIS.

Some of the other insights for treatment strategies are also interesting, although there are still some limitations because there is no direct evidence to support immunotherapy. At present, interventional embolization and surgery are the main treatments for DAVF, and some studies on stereotactic radiotherapy are also being carried out (142). However, there are still some lesions that cannot be obliterated due to anatomical reasons and material limitations. Meanwhile, the occurrence of *de novo* DAVF after treatment is also common (143–145). Furthermore, in addition to the elimination of lesions on imaging, the long-term prognosis of patients is also worthy of our attention. Therefore, innovative treatments are urgently needed. It is clear that the venous pressure, hypoxia, and inflammatory microenvironment do exist in the context of the lesion. As studies have shown, immunosuppressive therapy has potential in the treatment of CNS diseases (146). Factors of immunity may also be related to the prognosis of patients with this type of cerebrospinal vascular disease. As we found in our recent work, Complement component 4 binding protein alpha (C4BPA) and Complement Component 1, Q Subcomponent, A Chain (C1QA) are potential biomarkers for the diagnosis of spinal dural arteriovenous fistulas (SDAVFs), revealing that complement pathway activation might be one of the molecular mechanisms for venous hypertension myelopathy (147). Some clinical clues are worth pondering. There are many reports of confusion between SDAVF cases and myelitis cases (148). It is well known that steroids have good anti-inflammatory effects. However, hormonal shock therapy can lead to rapid deterioration of the patients’ symptoms. Notably, the use of hormones after surgery may be worth trying, which may improve patients’ prognosis. Importantly, given that immune factors play important roles in the overall disease process, it is really worth exploring whether boosting the beneficial immune factors or suppressing the detrimental immune factors can improve the problem.

## 5. Conclusion

In this review, we defined the MIS as a tertiary structure. Recent advances in meningeal immunity of healthy and diseased brains were reviewed. Moreover, by gaining clues from the immunity, we proposed new insights into the DAVF pathology, which may provide new ideas for understanding DAVF and looking for novel therapeutic targets. In conclusion, great breakthroughs have been made in the exploration of the potential functions of meninges, while there is still so much to be explored in this area that motivates our curiosity.

## Authors contributions

TT and ZP contributed equally to this work and should be considered to be co-first authors. TT reviewed related literatures and drafted the paper. ZP reviewed related literatures and drew the illustration figures. ZS checked for the literatures and contributed to the outline. YM discussed important points and made important revisions to the paper. HZ designed the concept and approved paper for publication. All authors contributed to the article and approved the submitted version.

## Funding

National Natural Science Foundation of China Award Number: 82101460.

## Acknowledgments

We thank Chengbin Yang M.D for his support of this study.

## Conflict of interest

The authors declare that the research was conducted in the absence of any commercial or financial relationships that could be construed as a potential conflict of interest.

## Publisher’s note

All claims expressed in this article are solely those of the authors and do not necessarily represent those of their affiliated organizations, or those of the publisher, the editors and the reviewers. Any product that may be evaluated in this article, or claim that may be made by its manufacturer, is not guaranteed or endorsed by the publisher.

## References

[1] Hartenstein V Giangrande A. Connecting the nervous and the immune systems in evolution. Commun Biol (2018) 1:64. doi: 10.1038/s42003-018-0070-2 30271946 PMC6123671

[2] Arbas EA Meinertzhagen IA Shaw SR. Evolution in nervous systems. Annu Rev Neurosci (1991) 14:9–38. doi: 10.1146/annurev.ne.14.030191.000301 2031578

[3] Ma T Wang F Xu S Huang JH. Meningeal immunity: Structure, function and a potential therapeutic target of neurodegenerative diseases. Brain Behav Immun (2021) 93:264–76. doi: 10.1016/j.bbi.2021.01.028 33548498

[4] Alves de Lima K Rustenhoven J Kipnis J. Meningeal immunity and its function in maintenance of the central nervous system in health and disease. Annu Rev Immunol (2020) 38:597–620. doi: 10.1146/annurev-immunol-102319-103410 32340575

[5] Louveau A. Meningeal immunity, drainage, and tertiary lymphoid structure formation. Methods Mol Biol (2018) 1845:31–45. doi: 10.1007/978-1-4939-8709-2_3 30141006

[6] Bell L Lenhart A Rosenwald A Monoranu CM Berberich-Siebelt F. Lymphoid aggregates in the CNS of progressive multiple sclerosis patients lack regulatory T cells. Front Immunol (2019) 10:3090. doi: 10.3389/fimmu.2019.03090 32010141 PMC6974514

[7] Zhang B Lang Y Zhang W Cui L Deng F. Characteristics and management of autoimmune disease-associated cerebral venous sinus thrombosis. Front Immunol (2021) 12:671101. doi: 10.3389/fimmu.2021.671101 34367137 PMC8339549

[8] Ghannam JY Al Kharazi KA. Neuroanatomy, cranial meninges. Treasure Island (FL: StatPearls (2021). 30969704

[9] Decimo I Fumagalli G Berton V Krampera M Bifari F. Meninges: from protective membrane to stem cell niche. Am J Stem Cells (2012) 1(2):92–105. 23671802 PMC3636743

[10] Lv X Wu Z Li Y. Innervation of the cerebral dura mater. Neuroradiol J (2014) 27:293–8. doi: 10.15274/NRJ-2014-10052 PMC4202893 24976196

[11] Mastorakos P McGavern D. The anatomy and immunology of vasculature in the central nervous system. Sci Immunol (2019) 4(37):eaav04924. doi: 10.1126/sciimmunol.aav0492 PMC6816468 31300479

[12] Louveau A Smirnov I Keyes TJ Eccles JD Rouhani SJ Peske JD. Structural and functional features of central nervous system lymphatic vessels. Nature (2015) 523:337–41. doi: 10.1038/nature14432 PMC4506234 26030524

[13] Herisson F Frodermann V Courties G Rohde D Sun Y Vandoorne K. Direct vascular channels connect skull bone marrow and the brain surface enabling myeloid cell migration. Nat Neurosci (2018) 21:1209–17. doi: 10.1038/s41593-018-0213-2 PMC6148759 30150661

[14] Veiga-Fernandes H Pachnis V. Neuroimmune regulation during intestinal development and homeostasis. Nat Immunol (2017) 18:116–22. doi: 10.1038/ni.3634 28092371

[15] Godinho-Silva C Cardoso F Veiga-Fernandes H. Neuro-immune cell units: A new paradigm in physiology. Annu Rev Immunol (2019) 37:19–46. doi: 10.1146/annurev-immunol-042718-041812 30379595

[16] Brioschi S Wang WL Peng V Wang M Shchukina I Greenberg ZJ. Heterogeneity of meningeal b cells reveals a lymphopoietic niche at the CNS borders. Science (2021) 373(6553):eabf9277. doi: 10.1126/science.abf9277 34083450 PMC8448524

[17] Cugurra A Mamuladze T Rustenhoven J Dykstra T Beroshvili G Greenberg ZJ. Skull and vertebral bone marrow are myeloid cell reservoirs for the meninges and CNS parenchyma. Science (2021) 373(6553):eabf7844. doi: 10.1126/science.abf7844 34083447 PMC8863069

[18] Savarin C Bergmann CC. Neuroimmunology of central nervous system viral infections: the cells, molecules and mechanisms involved. Curr Opin Pharmacol (2008) 8:472–9. doi: 10.1016/j.coph.2008.05.002 PMC2613975 18562249

[19] Pavlov VA Chavan SS Tracey KJ. Molecular and functional neuroscience in immunity. Annu Rev Immunol (2018) 36:783–812. doi: 10.1146/annurev-immunol-042617-053158 29677475 PMC6057146

[20] Fitzpatrick Z Frazer G Ferro A Clare S Bouladoux N Ferdinand J. Gut-educated IgA plasma cells defend the meningeal venous sinuses. Nature (2020) 587:472–6. doi: 10.1038/s41586-020-2886-4 PMC7748383 33149302

[21] Bartholomaus I Kawakami N Odoardi F Schlager C Miljkovic D Ellwart JW. Effector T cell interactions with meningeal vascular structures in nascent autoimmune CNS lesions. Nature (2009) 462:94–8. doi: 10.1038/nature08478 19829296

[22] Walsh DR Ross AM Newport DT Zhou Z Kearns J Fearon C. Mechanical characterisation of the human dura mater, falx cerebri and superior sagittal sinus. Acta Biomater (2021) 134:388–400. doi: 10.1016/j.actbio.2021.07.043 34314888

[23] Rustenhoven J Drieu A Mamuladze T de Lima KA Dykstra T Wall M. Functional characterization of the dural sinuses as a neuroimmune interface. Cell (2021) 184:1000–1016 e27. doi: 10.1016/j.cell.2020.12.040 33508229 PMC8487654

[24] Sandrone S Moreno-Zambrano D Kipnis J van Gijn J. A (delayed) history of the brain lymphatic system. Nat Med (2019) 25:538–40. doi: 10.1038/s41591-019-0417-3 30948855

[25] Ahn JH Cho H Kim JH Kim SH Ham JS Park I. Meningeal lymphatic vessels at the skull base drain cerebrospinal fluid. Nature (2019) 572:62–6. doi: 10.1038/s41586-019-1419-5 31341278

[26] Louveau A Herz J Alme MN Salvador AF Dong MQ Viar KE. CNS lymphatic drainage and neuroinflammation are regulated by meningeal lymphatic vasculature. Nat Neurosci (2018) 21:1380–91. doi: 10.1038/s41593-018-0227-9 PMC6214619 30224810

[27] Antila S Karaman S Nurmi H Airavaara M Voutilainen MH Mathivet T. Development and plasticity of meningeal lymphatic vessels. J Exp Med (2017) 214:3645–67. doi: 10.1084/jem.20170391 PMC5716035 29141865

[28] Ma Q Ineichen BV Detmar M Proulx ST. Outflow of cerebrospinal fluid is predominantly through lymphatic vessels and is reduced in aged mice. Nat Commun (2017) 8:1434. doi: 10.1038/s41467-017-01484-6 29127332 PMC5681558

[29] Absinta M Ha SK Nair G Sati P Luciano NJ Palisoc M. Human and nonhuman primate meninges harbor lymphatic vessels that can be visualized noninvasively by MRI. Elife (2017) 6:e29738. doi: 10.7554/eLife.29738 28971799 PMC5626482

[30] Jung E Gardner D Choi D Park E Jin Seong Y Yang S. Development and characterization of a novel Prox1-EGFP lymphatic and schlemm’s canal reporter rat. Sci Rep (2017) 7:5577. doi: 10.1038/s41598-017-06031-3 28717161 PMC5514086

[31] Shibata-Germanos S Goodman JR Grieg A Trivedi CA Benson BC Foti SC. Structural and functional conservation of non-lumenized lymphatic endothelial cells in the mammalian leptomeninges. Acta Neuropathol (2020) 139:383–401. doi: 10.1007/s00401-019-02091-z 31696318 PMC6989586

[32] Jacob L Boisserand LSB Geraldo LHM de Brito Neto J Mathivet T Antila S. Anatomy and function of the vertebral column lymphatic network in mice. Nat Commun (2019) 10:4594. doi: 10.1038/s41467-019-12568-w 31597914 PMC6785564

[33] Ringstad G Eide PK. Cerebrospinal fluid tracer efflux to parasagittal dura in humans. Nat Commun (2020) 11:354. doi: 10.1038/s41467-019-14195-x 31953399 PMC6969040

[34] Frederick N Louveau A. Meningeal lymphatics, immunity and neuroinflammation. Curr Opin Neurobiol (2020) 62:41–7. doi: 10.1016/j.conb.2019.11.010 31816570

[35] Da Mesquita S Louveau A Vaccari A Smirnov I Cornelison RC Kingsmore KM. Functional aspects of meningeal lymphatics in ageing and alzheimer’s disease. Nature (2018) 560:185–91. doi: 10.1038/s41586-018-0368-8 PMC6085146 30046111

[36] Aspelund A Antila S Proulx ST Karlsen TV Karaman S Detmar M. A dural lymphatic vascular system that drains brain interstitial fluid and macromolecules. J Exp Med (2015) 212:991–9. doi: 10.1084/jem.20142290 PMC4493418 26077718

[37] Iliff JJ Wang M Liao Y Plogg BA Peng W Gundersen GA. A paravascular pathway facilitates CSF flow through the brain parenchyma and the clearance of interstitial solutes, including amyloid beta. Sci Transl Med (2012) 4:147ra111. doi: 10.1126/scitranslmed.3003748 PMC3551275 22896675

[38] Chen J He J Ni R Yang Q Zhang Y Luo L. Cerebrovascular injuries induce lymphatic invasion into brain parenchyma to guide vascular regeneration in zebrafish. Dev Cell (2019) 49:697–710 e5. doi: 10.1016/j.devcel.2019.03.022 31006646

[39] Chen J Li X Ni R Chen Q Yang Q He J. Acute brain vascular regeneration occurs *via* lymphatic transdifferentiation. Dev Cell (2021) 56:3115–3127 e6. doi: 10.1016/j.devcel.2021.09.005 34562378

[40] Chen J He J Luo L. The brain vascular damage-induced lymphatic ingrowth is directed by Cxcl12b/Cxcr4a. Development (2022) 49(13):dev200729. doi: 10.1242/dev.200729 35694896

[41] Mundt S Mrdjen D Utz SG Greter M Schreiner B Becher B. Conventional DCs sample and present myelin antigens in the healthy CNS and allow parenchymal T cell entry to initiate neuroinflammation. Sci Immunol (2019) 4(31):eaau8380. doi: 10.1126/sciimmunol.aau8380 30679199

[42] Mundt S Greter M Flugel A Becher B. The CNS immune landscape from the viewpoint of a T cell. Trends Neurosci (2019) 42:667–79. doi: 10.1016/j.tins.2019.07.008 31474310

[43] Schlager C Korner H Krueger M Vidoli S Haberl M Mielke D. Effector T-cell trafficking between the leptomeninges and the cerebrospinal fluid. Nature (2016) 530:349–53. doi: 10.1038/nature16939 26863192

[44] Zhao Z Nelson AR Betsholtz C Zlokovic BV. Establishment and dysfunction of the blood-brain barrier. Cell (2015) 163:1064–78. doi: 10.1016/j.cell.2015.10.067 PMC4655822 26590417

[45] Yang AC Vest RT Kern F Lee DP Agam M Maat CA. A human brain vascular atlas reveals diverse mediators of Alzheimer's risk. Nature (2022) 603(7903):885–92. doi: 10.1038/s41586-021-04369-3 PMC9635042 35165441

[46] Wood H. VEGFA mediates blood-brain barrier disruption in Parkinson disease. Nat Rev Neurol (2022) 18:1. doi: 10.1038/s41582-021-00594-6 34789921

[47] Engelhardt B Vajkoczy P Weller RO. The movers and shapers in immune privilege of the CNS. Nat Immunol (2017) 18:123–31. doi: 10.1038/ni.3666 28092374

[48] Sweeney MD Sagare AP Zlokovic BV. Blood-brain barrier breakdown in Alzheimer disease and other neurodegenerative disorders. Nat Rev Neurol (2018) 14:133–50. doi: 10.1038/nrneurol.2017.188 PMC5829048 29377008

[49] Nation DA Sweeney MD Montagne A Sagare AP D’Orazio LM Pachicano M. Blood-brain barrier breakdown is an early biomarker of human cognitive dysfunction. Nat Med (2019) 25:270–6. doi: 10.1038/s41591-018-0297-y PMC6367058 30643288

[50] Kemp W.J.3RD Tubbs RS Cohen-Gadol AA. The innervation of the cranial dura mater: neurosurgical case correlates and a review of the literature. World Neurosurg (2012) 78:505–10. doi: 10.1016/j.wneu.2011.10.045 22120554

[51] Kimmel DL. Innervation of spinal dura mater and dura mater of the posterior cranial fossa. Neurology (1961) 11:800–9. doi: 10.1212/wnl.11.9.800 13756002

[52] Lee SH Shin KJ Koh KS Song WC. Visualization of the tentorial innervation of human dura mater. J Anat (2017) 231:683–9. doi: 10.1111/joa.12659 PMC5643920 28695607

[53] Fricke B Andres KH Von During M. Nerve fibers innervating the cranial and spinal meninges: morphology of nerve fiber terminals and their structural integration. Microsc Res Tech (2001) 53:96–105. doi: 10.1002/jemt.1074 11301485

[54] Witten A Marotta D Cohen-Gadol A. Developmental innervation of cranial dura mater and migraine headache: A narrative literature review. Headache (2021) 61:569–75. doi: 10.1111/head.14102 33749824

[55] Fricke B von During M Andres KH. Topography and immunocytochemical characterization of nerve fibers in the leptomeningeal compartments of the rat. a light- and electron-microscopical study. Cell Tissue Res (1997) 287:11–22. doi: 10.1007/s004410050728 9011385

[56] Andres KH von During M Muszynski K Schmidt RF. Nerve fibres and their terminals of the dura mater encephali of the rat. Anat Embryol (Berl) (1987) 175:289–301. doi: 10.1007/BF00309843 3826655

[57] Keller JT Marfurt CF. Peptidergic and serotoninergic innervation of the rat dura mater. J Comp Neurol (1991) 309:515–34. doi: 10.1002/cne.903090408 1717522

[58] Rice FL Xie JY Albrecht PJ Acker E Bourgeois J Navratilova E. Anatomy and immunochemical characterization of the non-arterial peptidergic diffuse dural innervation of the rat and rhesus monkey: Implications for functional regulation and treatment in migraine. Cephalalgia (2017) 37:1350–72. doi: 10.1177/0333102416677051 27852962

[59] Strassman AM Weissner W Williams M Ali S Levy D. Axon diameters and intradural trajectories of the dural innervation in the rat. J Comp Neurol (2004) 473:364–76. doi: 10.1002/cne.20106 15116396

[60] Davidson JR Mack J Gutnikova A Varatharaj A Darby S Squier W. Developmental changes in human dural innervation. Childs Nerv Syst (2012) 28:665–71. doi: 10.1007/s00381-012-1727-7 22395537

[61] McCulloch J Uddman R Kingman TA Edvinsson L. Calcitonin gene-related peptide: functional role in cerebrovascular regulation. Proc Natl Acad Sci U.S.A. (1986) 83:5731–5. doi: 10.1073/pnas.83.15.5731 PMC386363 3488550

[62] Bove GM Moskowitz MA. Primary afferent neurons innervating guinea pig dura. J Neurophysiol (1997) 77:299–308. doi: 10.1152/jn.1997.77.1.299 9120572

[63] Kosaras B Jakubowski M Kainz V Burstein R. Sensory innervation of the calvarial bones of the mouse. J Comp Neurol (2009) 515:331–48. doi: 10.1002/cne.22049 PMC2710390 19425099

[64] Kierdorf K Masuda T Jordao MJC Prinz M. Macrophages at CNS interfaces: ontogeny and function in health and disease. Nat Rev Neurosci (2019) 20:547–62. doi: 10.1038/s41583-019-0201-x 31358892

[65] Ding X Wang H Qian X Han X Yang L Cao Y. Panicle-shaped sympathetic architecture in the spleen parenchyma modulates antibacterial innate immunity. Cell Rep (2019) 27:3799–3807 e3. doi: 10.1016/j.celrep.2019.05.082 31242414

[66] Van Hove H Martens L Scheyltjens I De Vlaminck K Pombo Antunes AR De Prijck S. A single-cell atlas of mouse brain macrophages reveals unique transcriptional identities shaped by ontogeny and tissue environment. Nat Neurosci (2019) 22:1021–35. doi: 10.1038/s41593-019-0393-4 31061494

[67] Utz SG See P Mildenberger W Thion MS Silvin A Lutz M. Early fate defines microglia and non-parenchymal brain macrophage development. Cell (2020) 181:557–573 e18. doi: 10.1016/j.cell.2020.03.021 32259484

[68] Stromnes IM Cerretti LM Liggitt D Harris RA Goverman JM. Differential regulation of central nervous system autoimmunity by T(H)1 and T(H)17 cells. Nat Med (2008) 14:337–42. doi: 10.1038/nm1715 PMC2813727 18278054

[69] Pikor NB Astarita JL Summers-Deluca L Galicia G Qu J Ward LA. Integration of Th17- and lymphotoxin-derived signals initiates meningeal-resident stromal cell remodeling to propagate neuroinflammation. Immunity (2015) 43:1160–73. doi: 10.1016/j.immuni.2015.11.010 26682987

[70] Alves de Lima K Rustenhoven J Da Mesquita S Wall M Salvador AF Smirnov I. Meningeal gammadelta T cells regulate anxiety-like behavior *via* IL-17a signaling in neurons. Nat Immunol (2020) 21:1421–9. doi: 10.1038/s41590-020-0776-4 PMC8496952 32929273

[71] Kivisakk P Mahad DJ Callahan MK Trebst C Tucky B Wei T. Human cerebrospinal fluid central memory CD4+ T cells: evidence for trafficking through choroid plexus and meninges *via* p-selectin. Proc Natl Acad Sci U.S.A. (2003) 100:8389–94. doi: 10.1073/pnas.1433000100 PMC166239 12829791

[72] Derecki NC Cardani AN Yang CH Quinnies KM Crihfield A Lynch KR. Regulation of learning and memory by meningeal immunity: a key role for IL-4. J Exp Med (2010) 207:1067–80. doi: 10.1084/jem.20091419 PMC2867291 20439540

[73] Derk J Jones HE Como C Pawlikowski B Siegenthaler JA. Living on the edge of the CNS: Meninges cell diversity in health and disease. Front Cell Neurosci (2021) 15:703944. doi: 10.3389/fncel.2021.703944 34276313 PMC8281977

[74] Sato T Konishi H Tamada H Nishiwaki K Kiyama H. Morphology, localization, and postnatal development of dural macrophages. Cell Tissue Res (2021) 384:49–58. doi: 10.1007/s00441-020-03346-y 33433687

[75] Mitsdoerffer M Peters A. Tertiary lymphoid organs in central nervous system autoimmunity. Front Immunol (2016) 7:451. doi: 10.3389/fimmu.2016.00451 27826298 PMC5078318

[76] Lucchinetti CF Popescu BF Bunyan RF Moll NM Roemer SF Lassmann H. Inflammatory cortical demyelination in early multiple sclerosis. N Engl J Med (2011) 365:2188–97. doi: 10.1056/NEJMoa1100648 PMC3282172 22150037

[77] Wendeln AC Degenhardt K Kaurani L Gertig M Ulas T Jain G. Innate immune memory in the brain shapes neurological disease hallmarks. Nature (2018) 556:332–8. doi: 10.1038/s41586-018-0023-4 PMC6038912 29643512

[78] Ding XB Wang XX Xia DH Liu H Tian HY Fu Y. Impaired meningeal lymphatic drainage in patients with idiopathic parkinson’s disease. Nat Med (2021) 27:411–8. doi: 10.1038/s41591-020-01198-1 33462448

[79] Ennerfelt HE Lukens JR. The role of innate immunity in alzheimer’s disease. Immunol Rev (2020) 297:225–46. doi: 10.1111/imr.12896 PMC7783860 32588460

[80] Vijaya Kumar DK Moir RD. The emerging role of innate immunity in alzheimer’s disease. Neuropsychopharmacology (2017) 42:362. doi: 10.1038/npp.2016.226 27909333 PMC5143513

[81] Korin B Ben-Shaanan TL Schiller M Dubovik T Azulay-Debby H Boshnak NT. High-dimensional, single-cell characterization of the brain’s immune compartment. Nat Neurosci (2017) 20:1300–9. doi: 10.1038/nn.4610 28758994

[82] Schuchardt F Schroeder L Anastasopoulos C Markl M Bauerle J Hennemuth A. *In vivo* analysis of physiological 3D blood flow of cerebral veins. Eur Radiol (2015) 25:2371–80. doi: 10.1007/s00330-014-3587-x 25638218

[83] Roberg M Forsberg P Tegnell A Ekerfeldt K. Intrathecal production of specific IgA antibodies in CNS infections. J Neurol (1995) 242:390–7. doi: 10.1007/BF00868395 7561968

[84] Doss S Wandinger KP Hyman BT Panzer JA Synofzik M Dickerson B. High prevalence of NMDA receptor IgA/IgM antibodies in different dementia types. Ann Clin Transl Neurol (2014) 1:822–32. doi: 10.1002/acn3.120 PMC4241809 25493273

[85] Westman G Sohrabian A Aurelius E Ahlm C Schliamser S Sund F. Clinical significance of IgM and IgA class anti-NMDAR antibodies in herpes simplex encephalitis. J Clin Virol (2018) 103:75–80. doi: 10.1016/j.jcv.2018.04.007 29698873

[86] Rojas OL Probstel AK Porfilio EA Wang AA Charabati M Sun T. Recirculating intestinal IgA-producing cells regulate neuroinflammation *via* IL-10. Cell (2019) 177:492–3. doi: 10.1016/j.cell.2019.03.037 30951673

[87] Rua R McGavern DB. Advances in meningeal immunity. Trends Mol Med (2018) 24:542–59. doi: 10.1016/j.molmed.2018.04.003 PMC6044730 29731353

[88] Wood H. Microglial changes associated with meningeal inflammation in multiple sclerosis. Nat Rev Neurol (2021) 17:262. doi: 10.1038/s41582-021-00494-9 33846617

[89] van Olst L Rodriguez-Mogeda C Picon C Kiljan S James RE Kamermans A. Meningeal inflammation in multiple sclerosis induces phenotypic changes in cortical microglia that differentially associate with neurodegeneration. Acta Neuropathol (2021) 141:881–99. doi: 10.1007/s00401-021-02293-4 PMC8113309 33779783

[90] Mitsdoerffer M Di Liberto G Dotsch S Sie C Wagner I Pfaller M. Formation and immunomodulatory function of meningeal b cell aggregates in progressive CNS autoimmunity. Brain (2021) 144:1697–710. doi: 10.1093/brain/awab093 33693558

[91] Stower H. Meningeal lymphatics in aging and alzheimer’s disease. Nat Med (2018) 24:1781. doi: 10.1038/s41591-018-0281-6 30523314

[92] Da Mesquita S Papadopoulos Z Dykstra T Brase L Farias FG Wall M. Meningeal lymphatics affect microglia responses and anti-abeta immunotherapy. Nature (2021) 593:255–60. doi: 10.1038/s41586-021-03489-0 PMC8817786 33911285

[93] Yang AC Vest RT Kern F Lee DP Agam M Maat CA. A human brain vascular atlas reveals diverse mediators of alzheimer’s risk. Nature (2022) 603:885–92. doi: 10.1038/s41586-021-04369-3 PMC9635042 35165441

[94] Nguyen B Bix G Yao Y. Basal lamina changes in neurodegenerative disorders. Mol Neurodegener (2021) 16:81. doi: 10.1186/s13024-021-00502-y 34876200 PMC8650282

[95] Visanji NP Lang AE. Call the plumber: Impaired meningeal lymphatic drainage in parkinson’s disease. Mov Disord (2021) 36:1125. doi: 10.1002/mds.28590 33786905

[96] Zhou C Ma L Xu H Huo Y Luo J. Meningeal lymphatics regulate radiotherapy efficacy through modulating anti-tumor immunity. Cell Res (2022) 32:543–54. doi: 10.1038/s41422-022-00639-5 PMC9159979 35301438

[97] Hu X Deng Q Ma L Li Q Chen Y Liao Y. Meningeal lymphatic vessels regulate brain tumor drainage and immunity. Cell Res (2020) 30:229–43. doi: 10.1038/s41422-020-0287-8 PMC7054407 32094452

[98] Graham MS Mellinghoff IK. Meningeal lymphatics prime tumor immunity in glioblastoma. Cancer Cell (2021) 39:304–6. doi: 10.1016/j.ccell.2021.02.012 PMC9549925 33689701

[99] Fang Y Huang L Wang X Si X Lenahan C Shi H. A new perspective on cerebrospinal fluid dynamics after subarachnoid hemorrhage: From normal physiology to pathophysiological changes. J Cereb Blood Flow Metab (2022) 42:543–58. doi: 10.1177/0271678X211045748 PMC9051143 34806932

[100] Chen J Wang L Xu H Xing L Zhuang Z Zheng Y. Meningeal lymphatics clear erythrocytes that arise from subarachnoid hemorrhage. Nat Commun (2020) 11:3159. doi: 10.1038/s41467-020-16851-z 32572022 PMC7308412

[101] Tsai HH Hsieh YC Lin JS Kuo ZT Ho CY Chen CH. Functional investigation of meningeal lymphatic system in experimental intracerebral hemorrhage. Stroke (2022) 53:987–98. doi: 10.1161/STROKEAHA.121.037834 35144488

[102] Pedragosa J Salas-Perdomo A Gallizioli M Cugota R Miro-Mur F Brianso F. CNS-border associated macrophages respond to acute ischemic stroke attracting granulocytes and promoting vascular leakage. Acta Neuropathol Commun (2018) 6:76. doi: 10.1186/s40478-018-0581-6 30092836 PMC6083589

[103] Yanev P Poinsatte K Hominick D Khurana N Zuurbier KR Berndt M. Impaired meningeal lymphatic vessel development worsens stroke outcome. J Cereb Blood Flow Metab (2020) 40:263–75. doi: 10.1177/0271678X18822921 PMC7370617 30621519

[104] Bolte AC Lukens JR. Neuroimmune cleanup crews in brain injury. Trends Immunol (2021) 42:480–94. doi: 10.1016/j.it.2021.04.003 PMC8165004 33941486

[105] Bolte AC Dutta AB Hurt ME Smirnov I Kovacs MA McKee CA. Meningeal lymphatic dysfunction exacerbates traumatic brain injury pathogenesis. Nat Commun (2020) 11:4524. doi: 10.1038/s41467-020-18113-4 32913280 PMC7483525

[106] Watanabe R Kakizaki M Ikehara Y Togayachi A. Formation of fibroblastic reticular network in the brain after infection with neurovirulent murine coronavirus. Neuropathology (2016) 36:513–26. doi: 10.1111/neup.12302 PMC7167860 27121485

[107] Wilson EH Harris TH Mrass P John B Tait ED Wu GF. Behavior of parasite-specific effector CD8+ T cells in the brain and visualization of a kinesis-associated system of reticular fibers. Immunity (2009) 30:300–11. doi: 10.1016/j.immuni.2008.12.013 PMC2696229 19167248

[108] Hsu SJ Zhang C Jeong J Lee SI McConnell M Utsumi T. Enhanced meningeal lymphatic drainage ameliorates neuroinflammation and hepatic encephalopathy in cirrhotic rats. Gastroenterology (2021) 160:1315–1329 e13. doi: 10.1053/j.gastro.2020.11.036 33227282 PMC7956141

[109] Li X Qi L Yang D Hao S Zhang F Zhu X. Meningeal lymphatic vessels mediate neurotropic viral drainage from the central nervous system. Nat Neurosci (2022) 25:577–87. doi: 10.1038/s41593-022-01063-z 35524140

[110] Galeotti N Ghelardini C. Inhibition of the PKCgamma-epsilon pathway relieves from meningeal nociception in an animal model: an innovative perspective for migraine therapy? Neurotherapeutics (2013) 10:329–39. doi: 10.1007/s13311-012-0151-8 PMC3625380 23055050

[111] Erdener SE Kaya Z Dalkara T. Parenchymal neuroinflammatory signaling and dural neurogenic inflammation in migraine. J Headache Pain (2021) 22:138. doi: 10.1186/s10194-021-01353-0 34794382 PMC8600694

[112] Fontaine D Almairac F Santucci S Fernandez C Dallel R Pallud J. Dural and pial pain-sensitive structures in humans: new inputs from awake craniotomies. Brain (2018) 141:1040–8. doi: 10.1093/brain/awy005 29390108

[113] Burgos-Vega C Moy J Dussor G. Meningeal afferent signaling and the pathophysiology of migraine. Prog Mol Biol Transl Sci (2015) 131:537–64. doi: 10.1016/bs.pmbts.2015.01.001 25744685

[114] Wostyn P Van Dam D Audenaert K Killer HE De Deyn PP De Groot V. A new glaucoma hypothesis: a role of glymphatic system dysfunction. Fluids Barriers CNS (2015) 12:16. doi: 10.1186/s12987-015-0012-z 26118970 PMC4485867

[115] Tomczyk-Socha M Turno-Krecicka A. A novel uveolymphatic drainage pathway-possible new target for glaucoma treatment. Lymphat Res Biol (2017) 15:360–3. doi: 10.1089/lrb.2017.0019 29077522

[116] Soderman M Pavic L Edner G Holmin S Andersson T. Natural history of dural arteriovenous shunts. Stroke (2008) 39:1735–9. doi: 10.1161/STROKEAHA.107.506485 18388337

[117] van Dijk JM Terbrugge KG Willinsky RA Wallace MC. The natural history of dural arteriovenous shunts: the toronto experience. Stroke (2009) 40:e412; author reply e413-4. doi: 10.1161/STROKEAHA.108.545327 19342606

[118] Reynolds MR Lanzino G Zipfel GJ. Intracranial dural arteriovenous fistulae. Stroke (2017) 48:1424–31. doi: 10.1161/STROKEAHA.116.012784 PMC5435465 28432263

[119] Rustenhoven J Tanumihardja C Kipnis J. Cerebrovascular anomalies: Perspectives from immunology and cerebrospinal fluid flow. Circ Res (2021) 129:174–94. doi: 10.1161/CIRCRESAHA.121.318173 34166075

[120] Newton TH Cronqvist S. Involvement of dural arteries in intracranial arteriovenous malformations. Radiology (1969) 93:1071–8. doi: 10.1148/93.5.1071 5350675

[121] Yoon JY Regenhardt RW Leslie-Mazwi TM. Dural arteriovenous fistula presenting with reversible dementia. Ann Neurol (2021) 90:512–3. doi: 10.1002/ana.26138 34061386

[122] Ferro JM Coutinho JM Jansen O Bendszus M Dentali F Kobayashi A. Dural arteriovenous fistulae after cerebral venous thrombosis. Stroke (2020) 51:3344–7. doi: 10.1161/STROKEAHA.120.031235 32972315

[123] Koch MJ Stapleton CJ Guniganti R Lanzino G Sheehan J Alaraj A. Outcome following hemorrhage from cranial dural arteriovenous fistulae: Analysis of the multicenter international CONDOR registry. Stroke (2021) 52:e610–3. doi: 10.1161/STROKEAHA.121.034707 PMC8478891 34433307

[124] Terada T Tsuura M Komai N Higashida RT Halbach VV Dowd CF. The role of angiogenic factor bFGF in the development of dural AVFs. Acta Neurochir (Wien) (1996) 138:877–83. doi: 10.1007/BF01411267 8869717

[125] Uranishi R Nakase H Sakaki T. Expression of angiogenic growth factors in dural arteriovenous fistula. J Neurosurg (1999) 91:781–6. doi: 10.3171/jns.1999.91.5.0781 10541235

[126] Micieli JA Derkatch S Pereira VM Margolin EA. Development of dural arteriovenous fistulas after cerebral venous sinus thrombosis. J Neuroophthalmol (2016) 36:53–7. doi: 10.1097/WNO.0000000000000288 26214086

[127] Lindgren E Rentzos A Hiltunen S Serrano F Heldner MR Zuurbier SM. Dural arteriovenous fistulas in cerebral venous thrombosis: Data from the international cerebral venous thrombosis consortium. Eur J Neurol (2021) 29(3):761–70. doi: 10.1111/ene.15192 34811840

[128] Yu J Guo Y Wu Z Xu K. Traumatic arteriovenous fistula between the extracranial middle meningeal artery and the pterygoid plexus: A case report and literature review. Interv Neuroradiol (2017) 23:90–6. doi: 10.1177/1591019916673584 PMC5305156 27798326

[129] Vassilyadi M Mehrotra N Shamji MF Michaud J. Pediatric traumatic dural arteriovenous fistula. Can J Neurol Sci (2009) 36:751–6. doi: 10.1017/s0317167100008386 19960755

[130] Hamada Y Goto K Inoue T Iwaki T Matsuno H Suzuki S. Histopathological aspects of dural arteriovenous fistulas in the transverse-sigmoid sinus region in nine patients. Neurosurgery (1997) 40:452–6; discussion 456-8. doi: 10.1097/00006123-199703000-00005 9055283

[131] Wang SS Li CH Zhang XJ Wang RM. Investigation of the mechanism of dural arteriovenous fistula formation induced by high intracranial venous pressure in a rabbit model. BMC Neurosci (2014) 15:101. doi: 10.1186/1471-2202-15-101 25160131 PMC4152575

[132] Chen L Mao Y Zhou LF. Local chronic hypoperfusion secondary to sinus high pressure seems to be mainly responsible for the formation of intracranial dural arteriovenous fistula. Neurosurgery (2009) 64:973–83; discussion 983. doi: 10.1227/01.NEU.0000341908.48173.EB 19404157

[133] Shin Y Nakase H Nakamura M Shimada K Konishi N Sakaki T. Expression of angiogenic growth factor in the rat DAVF model. Neurol Res (2007) 29:727–33. doi: 10.1179/016164107X208077 17588308

[134] Zhu Y Lawton MT Du R Shwe Y Chen Y Shen F. Expression of hypoxia-inducible factor-1 and vascular endothelial growth factor in response to venous hypertension. Neurosurgery (2006) 59:discussion 687–96. doi: 10.1227/01.NEU.0000228962.68204.CF 16955051

[135] Yassari R Sayama T Jahromi BS Aihara Y Stoodley M Macdonald RL. Angiographic, hemodynamic and histological characterization of an arteriovenous fistula in rats. Acta Neurochir (Wien) (2004) 146:495–504. doi: 10.1007/s00701-004-0248-x 15118887

[136] Yamada M Yuzawa I Fujii K Miyasaka Y. Chronic cerebral venous hypertension model in rats. Neurol Res (2003) 25:694–6. doi: 10.1179/016164103101202174 14579784

[137] Lawton MT Jacobowitz R Spetzler RF. Redefined role of angiogenesis in the pathogenesis of dural arteriovenous malformations. J Neurosurg (1997) 87:267–74. doi: 10.3171/jns.1997.87.2.0267 9254092

[138] Herman JM Spetzler RF Bederson JB Kurbat JM Zabramski JM. Genesis of a dural arteriovenous malformation in a rat model. J Neurosurg (1995) 83:539–45. doi: 10.3171/jns.1995.83.3.0539 7666234

[139] Terada T Higashida RT Halbach VV Dowd CF Tsuura M Komai N. Development of acquired arteriovenous fistulas in rats due to venous hypertension. J Neurosurg (1994) 80:884–9. doi: 10.3171/jns.1994.80.5.0884 8169629

[140] Bederson JB Wiestler OD Brustle O Roth P Frick R Yasargil MG. Intracranial venous hypertension and the effects of venous outflow obstruction in a rat model of arteriovenous fistula. Neurosurgery (1991) 29:341–50. doi: 10.1097/00006123-199109000-00002 1922700

[141] Yang ST Rodriguez-Hernandez A Walker EJ Young WL Su H Lawton MT. Adult mouse venous hypertension model: common carotid artery to external jugular vein anastomosis. J Vis Exp (2015) 95:50472. doi: 10.3791/50472 PMC4354570 25650793

[142] Singh R Chen CJ Didwania P Kotecha R Fariselli L Pollock BE. Stereotactic radiosurgery for dural arteriovenous fistulas: A systematic review and meta-analysis and international stereotactic radiosurgery society practice guidelines. Neurosurgery (2022) 91:43–58. doi: 10.1227/neu.0000000000001953 35383682

[143] Duquette E Dowlati E Abdullah T Felbaum DR Mai JC Sur S. De Novo dural arteriovenous fistulas after endovascular treatment: Case illustration and literature review. Interv Neuroradiol (2022) 15910199221118517. doi: 10.1177/15910199221118517 35924383 PMC11475303

[144] Paramasivam S Toma N Niimi Y Berenstein A. *De novo* development of dural arteriovenous fistula after endovascular embolization of pial arteriovenous fistula. J neurointerv Surg (2013) 5:321–6. doi: 10.1136/neurintsurg-2012-010318 22510457

[145] Diana F Tosatto L Haznedari N Commodaro C Ruggiero M. *De novo* dural arteriovenous fistula on draining veins of previously treated pial arteriovenous malformation: a case report. J Stroke Cerebrovasc Dis (2021) 30:105798. doi: 10.1016/j.jstrokecerebrovasdis.2021.105798 33878548

[146] Correale J. Immunosuppressive amino-acid catabolizing enzymes in multiple sclerosis. Front Immunol (2021) 11:600428. doi: 10.3389/fimmu.2020.600428 33552055 PMC7855700

[147] Wang Y Ma Y Yang C Huang X Yang K Lan F. Potential biomarkers of spinal dural arteriovenous fistula: C4BPA and C1QA. J Neuroinflamm (2022) 19:165. doi: 10.1186/s12974-022-02522-x PMC9215050 35733178

[148] Murphy OC Hedjoudje A Salazar-Camelo A Pardo CA Gailloud P. Clinical characteristics, misdiagnosis and outcomes of patients with low-flow spinal arteriovenous fistulas. J Neurol Sci (2020) 413:116863. doi: 10.1016/j.jns.2020.116863 32386730

